# Disclosure to God as a Mediator Between Private Prayer and Psychological Well-Being in a Christian Sample

**DOI:** 10.1007/s10943-020-01107-3

**Published:** 2020-10-30

**Authors:** Beata Zarzycka, Dariusz Krok

**Affiliations:** 1 Institute of Psychology, The John Paul II Catholic University of Lublin, Al. Racławickie 14, 20-950 Lublin, Poland; 2 Institute of Psychology, Opole University, Opole, Poland

**Keywords:** Private prayer, Disclosure to God, Well-being

## Abstract

Although a number of studies have reported the psychological and physical benefits of prayer, only a few have examined the means by which prayer affects health. Winkeljohn Black et al. (J Relig Health 54(2):540–553, 2015. 10.1007/s10943-014-9840-4) found disclosure to God as a mediator in the relationship between prayer and mental health. In their study, the authors used Poloma and Pendleton’s (Rev Relig Res 31(1):46–53, 1989. https://doi.org/10.2307/3511023, ) model of prayer. This study examined whether disclosure to God as a mediator can be upheld with Laird et al.’s (Int J Psychol Relig 14(4):251–272, 2004) prayer model. The study included 285 Polish adults (50.2% of women), aged between 18 and 60 years. The Multidimensional Prayer Inventory, the Revised Distress Disclosure Index, and the Psychological Well-Being Scale were applied to the research. The results showed that the prayer of thanksgiving correlated positively and the prayer of supplication negatively with well-being. Two indirect effects were significant, indicating disclosure to God as a mediator of the confession—well-being link and the supplication—well-being link.

## Introduction

Private prayer, which is one of the indicators of religious faith, has been described as “the very soul and essence of religion” (James 1902, p. 361). Many authors have considered private prayer’s positive relationship to an individual’s mental health (Laird et al. 2004; Poloma and Pendleton 1991; Whittinton and Scher 2010). The research has documented positive correlates, including greater purpose in life, enhanced marital satisfaction, existential well-being (Laird et al. 2004), as well as negative correlates, including depression, anxiety, and drug use (Ladd and Spilka 2013). However, little work has been done to investigate what mechanisms explain prayer’s relationship with mental health (Pérez et al. 2011; Winkeljohn Black et al. 2015). A few constructs, for example perceived God mediated control (Jeppsen et al. 2015), trust-based beliefs in prayer (Pössel et al. 2014), internal dialogues (Puchalska-Wasyl and Zarzycka in print), rumination, and social support (Pérez et al. 2011) have been analyzed as potential mediators in the relationship between prayer and mental health. Winkeljohn Black et al. (2015) suggested disclosure to God as a mediator, which can explain prayer’s relationship with mental health, while tracking the type of prayer an individual most often uses. These mediating effects were analyzed in a Christian sample (Winkeljohn Black et al. 2015), and Jewish and Muslim samples (Winkeljohn Black et al. 2017). Winkeljohn Black et al.’s (2015, 2017) studies, using Poloma and Pendleton’s (1989) prayer types, found that disclosure to God mediated the relationship between colloquial and meditative prayers with mental health in a Christian sample. However, prayer is a complex, multidimensional construct, which has been described in many different ways. This study aimed to explore whether disclosure to God as a mediator can be upheld with Laird et al.’s (2004) conceptualization of prayer (Winkeljohn Black et al. 2015).

## Prayer Types

Laird et al. (2004) separated prayer into five types based on a historic Christian model of prayer known by the acrostic, ACTS: adoration, confession, thanksgiving, and supplication. Prayers of adoration are a type of prayer in which the focus is on the worship of God, without reference to specific circumstances or needs. Prayers of confession involve prayer in which faults, misdeeds, or shortcomings are acknowledged, and forgiveness is requested. Prayers of thanksgiving are expressions of gratitude towards God, made in reference to specific positive life circumstances. Supplication involves “requests for God’s intervention in specific life events for oneself or others” (Laird et al. 2004, p. 252). The authors added one additional type of prayer—reception—described in the literature as contemplative or receptive prayer. During reception prayer “one more passively awaits divine wisdom, understanding, or guidance” (Laird et al. 2004, p. 252).

Research has demonstrated that each of the prayer types have different associations with mental health. Adoration correlated positively with optimism and meaning in life (Whittinton and Scher 2010) and negatively with depressive symptoms (Pérez et al. 2011). Prayer of thanksgiving was a positive predictor of subjective well-being, self-esteem, and optimism (Whittinton and Scher 2010), and negative predictor of depression (Pérez et al. 2011). Supplication has a negative correlation with life satisfaction (Whittinton and Scher 2010). Prayer of reception was found to be a positive predictor of self-esteem, meaning in life, and optimism (Whittinton and Scher 2010) and a negative predictor of depressive symptoms (Pérez et al. 2011).

Prayer of confession was a negative predictor of subjective well-being, self-esteem, and optimism (Whittinton and Scher 2010). Prayer of supplication had a negative effect on subjective well-being (Whittinton and Scher 2010). Taken together, Whittinton and Scher (2010) suggest that there are two sets of prayer types, one which has a positive effect on well-being variables (thanksgiving, reception, and adoration), and the other which has a negative effect on well-being variables (confession and supplication). The models for the positive prayer types were generally confirmed, whereas the negative prayer types were not confirmed.

Winkeljohn Black et al. (2015) suggested that prayer types differ in the presence or quality of communication with God. For example, in Poloma and Pendleton (1991) colloquial prayer involves a more casual conversation with God than does ritual prayer, which involves more structured and formalized phrases. Making reference to the Laird et al.’s (2004) model, thanksgiving and confession prayers require communication with God to which people are praying. During thanksgiving prayer, the person engages in conversation with God expressing gratitude for life circumstances, whereas during confession prayer the person’s conversations with God involve acknowledging his/her faults, misdeeds, or shortcomings. Prayer of supplication involves requests for God’s interventions in specific life events for oneself or others. Laird’s supplication prayer type was very similar in content to Poloma and Pendleton’s (1989) petitionary prayer, which—as Winkeljohn Black et al. (2015) suggested—is less meaningfully relating to the person’s current affect or well-being. Adoration and reception do not include any communication with God. In the latter, the focus is on awaiting divine wisdom, understanding, or guidance from God, and in the former—on the worship of God, without reference to specific circumstances or needs. The differences in prayer types regarding the presence or quality of communication were considered within the context of Poloma and Pendleton’s (1991) study of prayer types’ association with mental health (Winkeljohn Black et al. 2015, 2017). The aim of this study was to examine whether these differences may affect prayer types’ association with well-being within the Laird et al.’s (2004) conceptualization of prayer.

## Disclosure to God as mechanism in the association of prayer and well-being

Results demonstrating the positive effects of private prayer on health raise the question of what mechanisms underlie these associations (Pössel et al. 2014). Winkeljohn Black et al. (2015) suggested that disclosure to God can be a mediator in the relationship between prayer and mental health. Self-disclosure is the communicative process of sharing personal thoughts, feelings, and experiences with another (Winkeljohn Black et al. 2015). Numerous studies have shown that one’s tendency to disclose personally distressing information is a predictor of psychological and physical well-being. Specifically, self-disclosure is positively associated with self-esteem, life satisfaction, positive emotions, and negatively with associated negative affect and physical well-being (for the review see Kahn et al. 2012).

Traditionally, self-disclosure is conceptualized as occurring between two or more persons. However, Bennett (2005) have conceptualized self-disclosure as occurring between a person and God (VandeCreek et al. 2002; Winkeljohn Black et al. 2015). Examples of such disclosures can be found in some Old Testament Psalms in which authors pour out their troubled thoughts and feelings before God. Also nowadays, many people disclose their problems, for example health concerns to God (VandeCreek et al. 2002). Winkeljohn Black et al. (2015, 2017) have begun investigating how prayers may include disclosures with the benefits to the discloser’s mental health in a Christian sample (Winkeljohn Black et al. 2015), as well as in Jewish and Muslim samples (Winkeljohn Black et al. 2017). They found that disclosure to God mediated the positive associations between colloquial and meditative prayer and mental health. Colloquial prayer involves talking to God in one’s own words while meditative prayer involves a noticing of the presence of God. However, disclosure to God mediates neither the association between petitionary prayer and mental health nor the relationship between ritual prayer and mental health. The latter involves written or prewritten prayers rather than the person’s wishes or thoughts, and the former does not involve self-reflection or candid conversation. Thus, the different types of prayer used may impact whether disclosure occurs for the person praying.

## Current study

The current study builds on previous work that has examined the association between different types of prayer and mental health. Using Laird et al.’s (2004) taxonomy of prayer types, we examined the association between psychological well-being and five types of prayer—adoration, confession, reception, supplication, and thanksgiving. Of particular importance, we examined whether disclosure to God, a known mediator between colloquial and meditative prayer types (Poloma and Pendleton 1989) and mental health among Christians (Winkeljohn Black et al. 2015), would mediate associations between Laird et al.’s (2004) prayer types and well-being. Because there have been no studies on disclosure to God in a Polish sample, findings similar to those of Winkeljohn Black et al. (2015) were expected.

It has been established that disclosing something to God during colloquial or meditative prayer is related to a decrease in psychological distress (Winkeljohn Black et al. 2015). Thus, it can be hypothesized that when an individual discloses something to God, he or she experiences an increase in psychological well-being. In Laird et al.’s (2004) model, confession and thanksgiving prayer types involve more personal communication with God. During confession an individual has the opportunity to thoughtfully disclose to God his/her faults, misdeeds, or shortcomings. Thanksgiving prayer involves expressions of gratitude for life circumstances. Therefore, it was hypothesized that the relationships of confession and thanksgiving prayer with well-being would be mediated by disclosure to God. Supplication involves requests for God’s interventions in specific life events for oneself or others. However, during supplication an individual does not have the opportunity to thoughtfully reflect on his/her life circumstances and disclose to God his/her needs (Winkeljohn Black et al. 2015). Thus, disclosure to God would not mediate the relationship between supplication prayer and well-being. Finally, adoration and reception, by definition, do not require communication with God. In adoration prayer, the focus is on the praise of God, without reference to specific circumstances or needs. In reception prayer, which is described as contemplative prayer, the focus is on awaiting divine wisdom, understanding, or guidance. Thus, it was predicted that there would be no significant relationships between adoration and reception and self-disclosure, and there would be either a positive or no relationship between adoration and receptive prayer and well-being. Following these predictions, it was hypothesized that self-disclosure would not mediate the relationship between adoration and reception prayer and well-being.

## Methods

### Respondents

The study included 285 adults (50.2% of women), aged between 18 and 60 years. Average age was 27.10 years (SD = 8.69). All the participants were Caucasians with Polish nationality. Most of them declared themselves as Catholics (*n* = 273, 95.8%); the other affiliations were as follows: Greek-Catholic (*n* = 3, 1.1%). Orthodox (*n* = 4, 1.4%), and Protestant (*n* = 5, 1.7%).

### Measures

#### Demographics

Participants were asked to indicate their age, gender, and religious affiliation. In addition, participants were asked to indicate their ethnic background, which was coded Caucasian or non-Caucasian.

#### Prayer Type

Prayer type was measured with the 21-item self-report, the Multidimensional Prayer Inventory (MPI, Laird et al. 2004). The items ask participants how often they engage in various prayer behaviors, with all items answerable on a 7-point Likert scale from 1 (*never*) to 7 (*all of the time*). The scale measures the frequency of behaviors for the five types of prayer: adoration, confession, thanksgiving, supplication, and reception. Example items for each prayer type are as follows, e.g., *I worshiped God* (adoration); *I confessed things that I had done wrong* (confession); *I offered thanks for specific things* (thanksgiving); *I made specific requests* (supplication); *I tried to be receptive to wisdom and guidance* (reception*).* The results of exploratory factor analysis confirmed the five-factor structure of the Polish MPI, consistent with the original scale. Together, all five factors accounted for 88.58% of the variance of the MPI. The internal consistency of the MPI obtained in this study was excellent (Table 2).

#### Disclosure to God

Participants completed the Revised Distress Disclosure Index (RDDI, Winkeljohn Black et al. 2017), a 12-item measure of participants’ tendency to disclose to God. The scale is a modified version of the Distress Disclosure Index (Kahn and Hessling 2001), which measures participants’ tendency to disclose versus conceal personally distressing information, thoughts, personal problems, and unpleasant emotions to another person (Kahn et al. 2012). The items were modified to measure one’s level of disclosure to God, e.g., *When I am in a bad mood, I pray about it*; *When I feel upset, I usually confide in God*. Items were statements rated on a five-point Likert response scale ranging from 1 (*strongly disagree*) to 5 (*strongly agree*). For the current study, exploratory factor analysis confirmed that the items load on a single factor, which accounted for 52.67% of the variance of the RDDI. The internal consistency for this sample was excellent (Cronbach’s alpha = .90).

#### Psychological Well-Being

The Psychological Well-Being Scale (PWBS) consists of 18 items reflecting the six areas of psychological well-being: autonomy, environmental mastery, personal growth, positive relations with others, purpose in life, and self-acceptance (Ryff 1989). Each aspect is represented by 3 items; example items for each domain are as follows: *I am not afraid to voice my opinions, even when they are in opposition to the opinions of most people* (autonomy); *In general, I feel I am in charge of the situation in which I live* (environmental mastery); *I like most aspects of my personality* (self-acceptance); *I have the sense that I have developed a lot as a person over time* (personal growth); *Most people see me as loving and affectionate* (positive relations); and *I have a sense of direction and purpose in life* (purpose in life). In the study, we used only the total score, which is a measure of overall well-being and is the mean of all the individual item scores. Respondents rated items on a scale ranging from 1 (*strongly disagree*) to 6 (*strongly agree*). The Polish adaptation of the PWBS was applied to the research (Cieciuch 2011). The internal consistency of the PWBS obtained in this study was satisfactory (Table 1).

**Table 1:** Descriptive statistics and partial correlations between the variables included in the study

	Adoration	Confession	Thanksgiving	Supplication	Reception	Disclosure	Well-being
Adoration	–						
Confession	.26***	–					
Thanksgiving	.45***	.09	–				
Supplication	− .05	.22***	.34***	–			
Reception	.31***	.25***	.13*	.29***	–		
Disclosure	.07	.01	.17**	.29***	.13*	–	
Well-being	.01	− .10	.20***	− .14*	.07	.13*	–
*M*	3.18	3.22	3.72	3.97	3.35	3.32	3.88
SD	1.70	1.65	1.83	1.84	1.67	.87	.42
Alpha	.94	.94	.95	.94	.90	.90	.79

*p < .05; **p <.01; ***p < .001

### Procedure

Participants were recruited by students of the undergraduate psychology program at the Catholic University of Lublin, as part of the course “Psychology of Religion”, which was taught by the first author in the 2018–2019 academic year. For their involvement in the study, the students were awarded credit points. No time constraints to complete the scales were imposed on the participants. The procedure was approved by the Research Ethics Committee at the Institute of Psychology at the first author’s university.

### Data analysis

The analyses were conducted in the following order. First, descriptive statistics (means, standard deviations, and alpha reliabilities) were calculated for all study variables. Next, to test the links between prayer types, disclosure to God, and well-being, correlational analyses were performed, partial correlations were calculated in order to control for shared variance among the variables. Thus, controlling for the shared variance among prayer types ensured that only the unique contribution of a prayer type’s variance to disclosure or psychological well-being was considered (Winkeljohn Black et al. 2015). Finally, to determine whether the associations between prayer types and well-being are mediated by disclosure to God, a series of mediation analyses were performed. Prior to testing the mediation models, raw scores were standardized on all variables (*M* = 0, SD = 1). Five separate mediation models were tested to assess direct and indirect effects of adoration, confession, thanksgiving, supplication, and reception on well-being mediated through disclosure to God. Age and sex were entered into the model as control variables, given that there is some literature indicating that these demographics influence disclosure and mental health (Winkeljohn Black et al. 2015). We performed all mediation analyses using SPSS’s adds-on Process, developed by Hayes (2018). Indirect effects and bias-corrected confidence intervals (CI 95%) were calculated using the bootstrapping procedure. Indirect effects were computed for each of 5000 bootstrapped samples.

## Results

Descriptive statistics and the Pearson partial correlations for each of the study variables are reported in Table 1. On the basis of kurtosis and skewness values, all variables had non-normal distributions. The mean scores on the Disclosure (− .598) and Well-Being (− .623) scales and the Supplication subscale (− .192) were slightly negatively skewed, with more high values. The mean score in the Adoration (.405), Thanksgiving (.037), Confession (.356), and Reception (.208) subscales were slightly positively skewed, with more low values. All the coefficients of skewness were less than one; thus, the skewness is not strong enough and can be ignored. The values of kurtosis are also within the acceptable range.

Two sets of correlational findings are particularly relevant to the purpose of the study. First, the three prayer types—thanksgiving, supplication, and reception—positively correlated with disclosure. Second, thanksgiving correlated positively and supplication correlated negatively with psychological well-being. Confession also correlated with well-being negatively, but this partial correlation was marginally insignificant (*p* = .09). Neither adoration nor confession correlated with disclosure to God. Adoration, confession, and reception did not correlate with well-being.

To explain the prayer types—well-being link, we conducted six mediation analyses in which adoration, confession, thanksgiving, supplication, and reception prayers were tested as predictors of well-being. Disclosure to God was tested as a mediator in the prayer type—well-being link. Both disclosure and the well-being were controlled for sex and age. Two indirect effects were significant, indicating disclosure to God as a mediator of the confession—well-being link and the supplication—well-being link (Table 2).

**Table 2:** 95% Confidence intervals for indirect effects through disclosure to God

	Effects	Lower CI	Upper CI
Adoration—PWB	.075	− .022	.173
Confession—PWB	.114	.031	.199
Thanksgiving—PWB	.033	− .076	.148
Supplication—PWB	.170	.055	.288
Reception—PWB	.082	− .012	.180

*PWB* psychological well-being

**Fig. 1:**
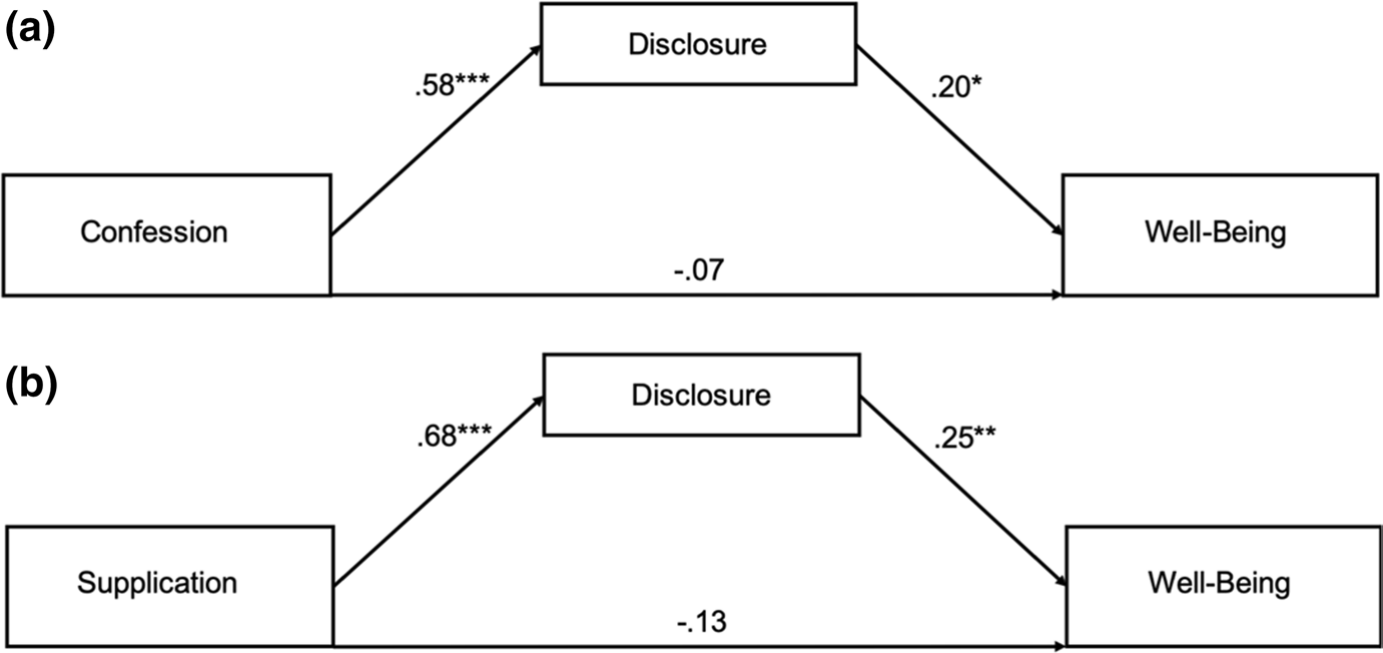
Standardized regression coefficients for the relationships between confession (**a**) and supplication (**b**) and well-being as mediated by disclosurse

As can be seen in Fig. 1a, disclosure to God fully mediated the relationship between prayer of confession and psychological well-being. The estimate for the indirect effect was .102 [.017, .179]. The standardized regression coefficient between the confession prayer and disclosure prayer was statistically significant, as was the standardized regression coefficient between disclosure and well-being. As shown in Fig. 1b, the same pattern of findings emerged with prayer of supplication and well-being. That is, disclosure to God mediated the relationship between prayer of supplication and psychological well-being. In the model examining prayer of supplication as a predictor, the estimate for the indirect effect was .154 [.037, .270]. The standardized regression coefficient between the supplication prayer and disclosure prayer was statistically significant, as was the standardized regression coefficient between disclosure and well-being. Thus, during confession and supplication prayer, people tend to disclose to God, which in turn increases their well-being. Neither confession nor supplication prayer was a significant predictor of psychological well-being after controlling for the mediator, implying that paths from prayer types to well-being were fully mediated by disclosure to God.

## Discussion

The objective of this study was to assess the mediating effect of disclosure to God in the relationship between prayer and well-being. Laird et al.’s (2004) five types of prayer were tested: adoration, confession, thanksgiving, supplication, and reception. The types of prayer revealed different patterns of associations with disclosure to God and well-being. Thanksgiving (thanks to God for specific positive outcomes), supplication (prayers asking God for specific things), and reception (prayers focused on opening oneself up to closeness to God) were found to be positively associated with disclosure to God. Prayers of thanksgiving were significantly associated with higher levels of well-being, whereas prayers of supplication were significantly associated with lower levels of well-being. Consistent with the hypothesis and previous literature (Winkeljohn Black et al. 2015, 2017), disclosure to God was confirmed as a mediator in the prayer—well-being link. More specifically, results from mediation analyses indicated that disclosure to God mediated the relationship between confession prayer (prayers admitting one’s sins to God) and psychological well-being. However, contrary to our hypothesis, disclosure to God did not mediate the relationship between thanksgiving prayer and well-being, but it mediated the relationship between supplication prayer and well-being. Thus, during confession and supplication prayer people disclose to God, which in turn strengthens their well-being. These findings support the claim that disclosure to God explains the unique mechanism between different prayer types and well-being (Pérez et al. 2011; Winkeljohn Black et al. 2015, 2017).

The first objective of the study was to assess the relationships between prayer types and well-being. Although the present study found that two types of prayer—thanksgiving and supplication—were significant predictors of well-being, it is worth noting that thanksgiving correlated with well-being positively, whereas supplication—negatively. During thanksgiving prayer, the individuals who are praying are focused on God and on what they have received from God, which in turn enhances their well-being. Perhaps this finding should not come as too great a surprise as Whittinton and Scher (2010) confirmed a positive effect of thanksgiving on well-being. Thanksgiving prayer was also associated with subjective well-being in a sample of patients with osteoarthritis (Laird et al. 2004) and in cancer patients (Pérez et al. 2011). Supplication prayer is aimed at getting something from God and requires the person praying to focus on what they are lacking or on his/her unfulfilled needs. Thus, as this study suggested, supplication correlated with well-being negatively, which is in line with what Whittinton and Scher (2010) suggest that supplication belongs to a set of prayer types, which has a negative effect on well-being variables.

Some types of prayer were not associated with well-being in our sample. For example, there were no relationships between adoration and reception with well-being. Little research has been conducted on these two types of prayer (Pérez et al. 2011). Whittinton and Scher (2010) claimed that both adoration and reception prayer belong to a set of prayer types that positively affect well-being variables. Pérez et al. (2011) also showed that adoration was associated with lower depressive symptoms in a sample of cancer patients. However, Laird et al. (2004) observed that adoration was not associated with well-being among patients with either rheumatoid arthritis or osteoarthritis (Laird et al. 2004). The reason for these inconsistencies can be because the researchers included different, more specific measures of well-being, such as satisfaction with life, self-esteem, optimism, meaning of life, and depression that showed different patterns of correlations with prayer types. Psychological well-being is rather a general measure, which might not differentiate these specific patterns of correlations.

An important contribution of this study is the examination of potential mechanisms that may explain the Laird et al. (2004) prayer types—well-being link. The results of this study suggest that psychological well-being may be enhanced for people who offer prayers of confession or supplication, because these types of prayer increase disclosure to God. People who during their prayer focus on their faults, misdeeds, shortcomings, or on what they are lacking, may more easily open their thoughts, feelings, and emotions before God, which may lead to an increase in their well-being. It is possible that confession and supplication prayers cultivate well-being, by providing an opportunity to communicate with God in a more personal way.

The correlation analysis revealed that both supplication and confession (the latter marginally insignificant) prayers were associated to lower levels of well-being. However, after controlling disclosure to God as a mediator, neither confession nor supplication prayer was a significant predictor of psychological well-being. Thus, awareness of their own shortcomings or unfulfilled needs or desires may foster well-being, when people who are praying disclose their thoughts and emotions to God. This result is only partially in line with the results by Winkeljohn Black et al. (2015), in which disclosure to God did not mediate the relationship between petitionary prayer and mental health. Thus, although supplication and petitionary prayers can be considered very similar in content (Whittinton and Scher 2010; Winkeljohn Black et al. 2015), they seem not to be identical. Supplication literally means *a request* or *petition*. However, in petitionary prayer, the person who is praying initiates communication by asking God for a material item. Supplication involves requests for God’s intervention in specific life events for oneself or others. In a spiritual context, a person who makes supplication humbly presents his/her requests before God—with an expectation that God will answer them. This can suggest that during supplication prayer, people relate to their inner needs when they express them, which in turn can influence the level of their well-being.

A direct association between prayer of thanksgiving and well-being remained significant, which may suggest that this link is likely mediated by additional factors. It would be useful for future studies to examine other variables, for example positive affect, as potential mechanisms in the relationship between thanksgiving and well-being (Pérez et al. 2011).

In regards to shortcomings of the study, it should be emphasized that the cross-sectional, non-experimental design limits our ability to determine the causal direction that explains the relationship between the prayer types and well-being. A reverse causal direction is not impossible, for example those with lower psychological well-being may be more likely to engage in supplication prayer (Whittinton and Scher 2010). Experimental studies are warranted in order to determine if the relationship between type of prayer and well-being is causal. Second, the study was based on individuals’ self-reports, and thus, response bias could not be controlled. However, this possibility may be tempered somewhat by the fact that respondents completed the measures anonymously. Third, participants in this study were a non-random sample of predominantly White, well-educated, Polish Roman Catholic Christians. Therefore, it is difficult to generalize the results of this study to populations that differ by race, socioeconomic status, and religious affiliation. The results need to be replicated with samples where the current shortcomings are minimized.

In spite of these limitations, this study makes some important contributions. First of all, two prayer types—thanksgiving, and supplication—have been found to be correlates of psychological well-being. However, thanksgiving correlated positively and supplication negatively with well-being. Second, disclosure to God has been confirmed as a mediator in the relationship between prayer types with well-being. As predicted, disclosure to God mediated the relationship between confession prayer and well-being. However, contrary to our expectation, disclosure to God also mediated the relationship between supplication prayer and well-being. Third, the latter result, which is not in line with Winkeljohn Black et al.’s (2015) results, raises the issue of whether there is a difference between supplication (Laird et al. 2004) and petitionary (Poloma and Pendleton 1989) prayer. Finally, the present findings also add new evidence from the Polish, mostly Catholic population, on the prayer types—well-being link in the light of the fact that the majority of the previous studies were conducted in Western, mostly American, samples.
